# Does the Missing Data Imputation Method Affect the Composition and Performance of Prognostic Models?

**Published:** 2012-01-01

**Authors:** M R Baneshi, A R Talei

**Affiliations:** 1 Research Center for Modelling in Health, Kerman University of Medical Sciences, Kerman, Iran; 2 Shahid Faghihi Hospital, Shiraz University of Medical Sciences, Shiraz, Iran

**Keywords:** Data, Multivariable imputation via chained equations, Expectation maximum algorithm, Breast cancer

## Abstract

**Background:**

We already showed the superiority of imputation of missing data (via Multivariable Imputation via Chained Equations (MICE) method) over exclusion of them; however, the methodology of MICE is complicated. Furthermore, easier imputation methods are available. The aim of this study was to compare them in terms of model composition and performance.

**Methods:**

Three hundreds and ten breast cancer patients were recruited. Four approaches were applied to impute missing data. First we adopted an ad hoc method in which missing data for each variable was replaced by the median of observed values. Then 3 likelihood-based approaches were used. In the regression imputation, a regression model compared the variable with missing data to the rest of the variables. The regression equation was used to fill the missing data. The Expectation Maximum (E-M) algorithm was implemented in which missing data and regression parameters were estimated iteratively until convergence of regression parameters. Finally, the MICE method was applied. Models developed were compared in terms of variables significantly contributed to the multifactorial analysis, sensitivity and specificity.

**Results:**

All candidate variables significantly contributed to the MICE model. However, grade of disease lost its effect in other three models. The MICE model showed the best performance followed by E-M model.

**Conclusion:**

Among imputation methods, final models were not the same, in terms of composition and perform­ance. Therefore, modern imputation methods are recommended to recover the information.

## Introduction

The problem of missing data arises in majority of medical data sets[1] An ad hoc method was shown to substitute missing data by a fixed value such as the mean (in the case of normally distributed data) or median of observed values (in the case of skewed data). This approach might artificially reduce the variance and affect the strength of relationships with other variables since all missing data are replaced by a single value.[2][3][4] Furthermore, in case-control studies, replacement of missing data with a fixed value in­creases the overlap between cases and controls and tends to underestimate the true association.[5]

Recent developments in the field of analysis of missing data, including Expectation Maximum (EM) algorithm and Multiple Imputation via Chained Equa­tions (MICE), provided methods to deal with missing data adequately. These methods are likelihood based and use partially observed data to impute incomplete data. Although such methods provide better estimates, computer software skills are required and communica­tion of methods with clinical audiences might not be simple. It has been noted that 'the optimal method should balance validity, ease of interpretability for readers and analysis expertise of the research team.[6]

We already used a breast cancer data set to illus­trate different methodological issues.[7][8][9] In a recent work, we addressed the process of the MICE method and its superiority over the complete case analysis.[10]The philosophy behind the MICE model is not simple especially to clinical audience. On the other hand, easier imputation methods are available. There is evi­dence that, although easier methods involve some limitations, they might provide similar model to that of the MICE under special circumstances.

In this article, we first reviewed studies published in the literature to understand the situations in which easier methods worked as a good approximation for sophisticated methods. Then main aim of this study was to apply imputation methods and to compare them in terms of variables remain in the final model, sensitivity, and specificity. In addition, results were compared with that of the MICE.[10]

## Materials and Methods

A total of 310 breast cancer patients formed the study population. Data were collected from Hospital-based cancer registry of Nemazee Hospital, affiliated to Shiraz University of Medical Sciences. The main out­come of study was Breast Cancer Specific Death (BCSD). Candidate variables for the multifactorial models were tumor stage with 3 levels (early, locally advanced, and advanced), tumor grade with 3 levels (1, 2 and 3), history of benign breast disease (positive versus negative), and age at diagnosis.[7][9][11][12]

Totally, 4 models were developed. The only dif­ference between them was the approach utilised to deal with missing data (see below). In development of all models, Cox regression model was fitted to the data. Since none of the variables under study was continuous, no attempt was made to optimise the form of association between variables and BCSD.[13]In other words, no form of polynomial regression was needed. Models developed were as follows.

In the median model, for each variable, missing data were replaced by the median of observed values. For example stage variable had three categories 1, 2 and 3. Patients with an unknown stage were ignored, and the median of stage variables for the rest of pa­tients was calculated. Unknown stages were then simply filled by the median.

In regression model, each variable including miss­ing data, in turn, was considered as dependant vari­able, using other variables as independent ones. A linear regression model was then fitted only using cases with available data on all variables. For exam­ple, a regression model linked stage to grade, age, status, and history of benign disease. Missing data were estimated from model derived, following a rounding approach to the nearest plausible value. For example, for stage and grade, which had 3 levels, im­puted values <1.5 were rounded to 1. Furthermore, values higher than 2 but lower than 2.5 were rounded to 2. For family history of benign form of disease and age at diagnosis, which had 2 levels, imputations <1.5 were rounded to 1 and values >1.5 were rounded to 2.

The E-M model was the extension of the regression model. Here, using cases with available data, regression models would be fitted. This model was used to impute the missing data. Then regression parameters would be re-estimated using new sets of data (i.e. using cases with available data plus imputed data). Again, new parameters of regression model (i.e. coefficients) would be estimated. Using ew regression coefficients, imputed values would be updated. These two steps would be continued iteratively until convergence. The whole process would be stopped when difference between regression coefficients in two successive steps became less than 10-6. Although it is necessary that data follow a multivariate normal distribution, it was suggested that for binary and categorical data, a rounding approach to the nearest possible value might work well in practice.[14]

Regression and E-M imputations methods replace each missing data by one single value. On the other hand, the MICE method was taken into account for the imputation uncertainty. Methodological issues behind the MICE model were illustrated elsewhere.[10]The key concept of the MICE method was to use the distribution of the observed data to estimate plausible values for the missing data and to incorporate random components into the estimated values so as to reflect their uncertainty. Here, each missing data was re­placed by 10 values, thus creating 10 imputed data sets.[15] Each data set was analysed individually but e.13stimates derived from imputed data sets were combined applying Rubin's rule, to get a single Haz­ard Ratio (HR) and Confidence Interval (CI).[16]

Models developed were compared in terms of variables contributed significantly to them and esti­mated HR's. To compare the sensitivity and specific­ity of models, in each model, linear combination of variables multiplied into the estimated regression co­efficients was calculated (i.e. risk score). Risk scores were dichotomised at median to divide patients into good and bad prognosis groups. Two by two tables were constructed using prognostic groups and patients' status. Sensitivity and specificity were calcu­lated with respect to patients' status.

A series of packages which worked under R software(version 2.5.1) were used.[17] Missing data were imputed using MICE package.[18] Estimated regression coeffi­cients and standard errors were combined across im­puted data sets using Mitools library.[19] The E-M and regression imputations were done using SPSS software.

## Results

Total number of BCSD was 56 (out of 310 patients). The numbers (percentages) of patients with missing value were as follows: Node status 63 (20.3%), grade 64 (20.6%), history of benign disease 47 (15.2%), and age at diagnosis 0 (0%).

In terms of variables contributed o the models, all four variables were significant in the MICE model (Table 1). However, in the E-M and median models, grade of disease lost its significant impact on BCSD. This was the case for age of diagnosis as well. Re­gression model produced poorest result. In the MICE model, the risk of death for patients with high stage was about 3 times higher than that of patients with low stage. This variable was of marginal significance in the regression model. On the other hand, the age variable was retained in the regression model.

**Table 1:** Comparison between imputation models in terms of composition and performance.

**Variable**	**Level**	**Median model**		**Regression model**		**E-M model**		**MICE model**	
		**HR^a^ (95% CI^b^)**	***P*value**			**HR^a^ (95% CIb)**	***P*value**	**HR^a^(95% CI^b^)**	***P*value**
Stage	1	1		1		1		1	
	2	3.79 (1.96, 7.33)	<0.001	2.57 (1.39, 4.75)	0.003	3.84 (1.94, 7.22)	<0.001	3.13 (1.64, 5.97)	<0.001
	3	2.99 (1.24, 7.13)	0.014	2.17 (0.94, 4.99)	0.07	3.21 (1.35, 7.65)	0.01	2.53 (1.05, 6.12)	0.03
Grade	1	1		1		1		1	
	2	1.69 (0.82, 3.49)	0.16	2.03 (1, 4.10)	0.05	1.56 (0.76, 3.19)	0.22	2.46 (1.15, 5.24)	0.02
	3	1.25 (0.56, 2.80)	0.59	1.51 (0.67, 3.37)	0.32	1.27 (0.56, 2.84)	0.57	1.52 (0.65, 3.60)	0.34
Age	<48	1		1		1		1	
	≥48	1.80 (0.95, 3.45)	0.07	2.12 (1.41, 3.95)	0.02	1.72 (0.89, 3.32)	0.11	1.92 (1.01, 3.65)	0.04
Benign	No	1		1		1		1	
	Yes	2.26 (1.25, 4.11)	0.01	2.29 (1.27, 4.13)	0.01	2.13 (1.15, 3.94)	0.02	2.32 (1.24, 4.33)	0.01
Performance of models									
Sensitivity		23%		36%		88%		88%	
Specificity		59%		48%		56%		60%	

^a^ HR: Hazard ratio

^b^ CI: Confidence interval, SD: Standard deviation

In terms of performance, the MICE model was able to classify 49 died patients (out of 56) into bad progno­sis group giving sensitivity of 0.88. Furthermore, out of 254 live patients, 151 ones were allocated to good prognosis group. This was corresponded to a specific­ity of 0.60. Results of he E-M model were fairly the same as MICE. However, regression and median sub­stitution models produced poorest results. Sensitivity and specificity of these two models were 0.36 and 0.23 respectively. Corresponding figure for specificity was 0.48 and 0.59 respectively.

## Discussion

Using an empirical data set, our results showed that different imputation methods led to different models in terms of composition and performance. It should be emphasized that we did not aim to perform com­plicated simulation studies, so as to study behaviour of imputation methods under different circumstances. We simply tried to show the impact of imputation method on modelling. This provided an excellent stage to explain to clinicians why different methods to handle missing data yielded different results.

In our data set, the MICE model gave highest sen­sitivity and specificity and retained all variables as being significant (Table 1). However, the communi­cation of results with clinical audiences was not sim­ple and especial software was required (Table 2).

**Table 2:** Advantages and disadvantages of methods to tackle missing data.

**Feature**	**Complete-case**	**Median substitution**	**EM**	**MICE**
No special software is needed	Yes	Yes	Yes	No
Easy to communicate with clinical audience	Yes	Yes	Yes	No
Do not require distributional assumption	Yes	Yes	No	Yes
Preserve data characteristics	No	No	Yes	Yes
Convergence of imputation model is not an issue	Yes	Yes	No	No
Takes imputation uncertainty into account	No	No	No	Yes
Any particular problem	Diminishes the power Gives biased estimated if not MCAR	Artificially reduces the variance	Might give out of range estimates	Requires aggregation of estimates

We have seen that results of the median model were not comparable with that of MICE. Neither composition of model nor performance was satisfy­ing. It has been argued that although replacement of missing data with a single value does not hold charac­teristics of data, this ad hoc method is reasonable when missing rate is low.[20][21][22][23][24] Substitution of missing data by median of observed values was straightfor­ward. However, in our data set, this model was only able to identify less than 40% of died patients. The specificity rate was less than 60%.

Results of the regression model were disappointing as well. Stage which was one of the most important prognostic factors of breast cancer was of marginal significance in this model. Furthermore, sensitivity and specificity of the model was as poor as that of median replacement model. This is because the effect of stage, which is known as one of the most important risk factor[20][21] did not reach a significant level.

The main disadvantage of EM method was that uncertainty in imputation of missing data was not taken into account. Furthermore, although EM pre­serves characteristics of data set[25] but might fail to converge when sample size relative to number of variables was low. In our application, performance of models derived applying EM and MICE methods were the same in terms of performance but not in terms of contribution of variables in the model. Age at diagnosis was not retained in the E-M model but it did not affect the performance of the model. This might indicate low contribution of this variable in terms of classification of patients.

With our experience, the main advantages and disadvantages of imputation methods were gathered in Table 2. Each technique had its own limitations. For example, median replacement was simple to be im­plemented but underestimated the true association. Our results suggested that the MICE method resulted into the best composition and performance. However, our findings can be considered only as a case study. This is because several items (such as sample size, nature of variables, and rate of missing data) affect performance of imputation models (see limitations of our work in the rest of the text). Therefore, here we reviewed the literature to compare our findings with them.

In a comprehensive study, it has been shown that the MICE method was the best technique to deal with missing data (see rest of the text).[26] However, there is controversy in the appropriateness of replacement of missing data by a fixed value. Usefulness of ad hoc methods, such as replacement with a fixed value, de­pends to a great extent to missing rate and content. Here, some examples were presented.

As an example, performance of complete-case analysis (C-C), mean replacement, and MICE imputa­tion methods were compared.[23] Models were com­pared in terms of magnitude of estimated coefficients and standard error (SE), direction of association, and discrimination ability. Data for 398 cases with sus­pected pulmonary embolism were available of which 246 participants (62%) had complete information on all 26 variables studied. Rate of missing values were as follows: 0% for12 variables, <10% for 11 variables 14% for 1 variable and 21% for 2 variables. Variables selected in the complete-case analysis differed with other methods. Results of MICE were comparable with mean replacement because of low overall num­ber of missing values. As expected, replacement of missing data by mean yielded smaller SE's.

On the other had, to address replacement of missing data by a single value was a bad practice, a total of 1000 samples of 500 subjects were generated.[5] True odds ra­tio (OR) between diagnostic test and disease status was 2.7. Omitting data under MCAR mechanism, diagnostic test for 20% of diseased and non-diseased subjects were omitted. When missing data were then replaced with overall mean, estimated OR was 1.73.

In another study, using a data set of a study on substance use among American Indian adolescents, artificial reduction in SE in the case of replacement of missing data by a single value was addressed.[4] In the original paper, 76% of cases with available data were analysed.[27] This gave mean at first use (SE) of 14.66 (0.19). Use of E-M or substitution of missing data by mean reduced SE to 0.16.

A sample of 492 patients with complete data, from a longitudinal study on the stress and health of elder adults was used to address the ability of list-wise de­letion, mean substitution, and EM algorithm to handle missing data.[25] True mean (SD) for all cases (n=492) was 7.34 (7.28). Data on a single variable for 96 cases were dropped out (missing rate of 20%) under MAR assumption. Estimates corresponding to C-C and mean substitution were 6.47 (6.82) and 6.38 (6.12). EM gave best estimate of 6.79 (6.23). Furthermore, after application of EM method, correlation between variable with missing value and rest of variables were fairly similar to that of original data.

In a comprehensive study with binary outcomes, the ability of mean replacement, MI techniques, and complete case analysis were compared.[26] The data set of Society of Cardiothoracic Surgeons of Great Brit­ain and Ireland was used. The original data included 20378 cases of which 1404 patients had died. Nine variables, out of 14 variables studied involved less than 15% missing values. The missing rate for 3 vari­ables was higher than 20%. Actual missing rates were 20.2%, 42.7%, and 53.4%. In total, 32% had com­plete data on all variables studied. Authors found that, under MAR and MCAR mechanisms, performance of the MICE and conditional mean substitution were comparable and much better than complete-case analysis in terms of the proportion of patients classi­fied into the correct risk group and the estimated spearman rank correlation between true and fitted probabilities. Estimated root mean square error (which quantified difference between fitted and true probabilities) for the mean substitution method was marginally higher than that of the MICE but better than complete-case analysis. Comparing estimated regression coefficients, it has been shown that the MICE produced the lowest level of bias.[26]

Application of ad hoc methods such as replacement with mean was criticised.[4] This might lead to artificially narrow confidence intervals. However, it has been sug­gested that when missing rate is low (about 10%), re­placement of missing data by median or mean or exclu­sion of missing data is a reasonable approximation for the MICE, in terms of variables that contribute to the multifactorial models. However, such ad hoc methods affect estimated HR's and model performance.

The main limitations of our work were as follows. We used a data set contained only four categorical variables. Therefore questions whether number and nature of variables affected our conclusions remains to be addressed. In regression imputation, E-M algo­rithm, and the MICE methods regression modelling has been used to draw the imputation. It is clear that, generally speaking, the more the number of variables the better the prediction. In addition, in regression and E-M imputations, it is assumed that the data fol­lows a normal distribution. This was not the case in our data set. This might partly explain poorer per­formance of these two methods. Another limitation of our work was that we did not compare performance of the imputation methods under different missing rates. One last issue was the process of model build­ing. We developed all four models using ENTER method. This method fits a model including all vari­ables offered to. Therefore, the behavior of imputa­tion models under different variable selection meth­ods (i.e. Backward and Forward) should be investi­gated. All issues noted affect the conclusions[5][25][26][27][28] and their influence should be explored in future studies.

Although the MICE method does not provide unique estimates,[29] and communication of results with clinical audiences is not simple, evidence from litera­ture suggested that the MICE method is the best ap­proach to impute missing data. However, our literature review showed that under special circumstances, easier methods might provide comparable estimates. It should be noted that even when easier imputation methods work, results should be compared to that of MICE, to enrich the body of the literature and enhance the un­derstanding of the value of the statistical methods.
